# Supplementary material for: Distribution of bacteria and antimicrobial resistance in retail Nile tilapia (Oreochromis spp.) as potential sources of foodborne illness
Source: PLoS One. 2024 Apr 2;19(4):e0299987. doi: 10.1371/journal.pone.0299987 (PMC10986973; doi:10.1371/journal.pone.0299987)
Supplement: S1 Table — (DOCX) [file pone.0299987.s001.docx]

**S1 Table. Primers used for virulence gene detection of *A. hydrophila*, *Salmonella* spp., and *V. cholerae*.**

| **Gene** | **Primer** | **Oligonucleotide sequences** | **Product size (bp)** | **Reference** |
| --- | --- | --- | --- | --- |
| ***A. hydrophila*** | |  |  |  |
| *aer* | *aer*-F | CTACTTTTGCCGGCGAGCGG | 953 | [[26](#ref26)] |
|  | *aer*-R | TGATTCCCGAAGGCACTCCC |  |  |
| *ah* | *ah*-F | GAAAGGTTGATGCCTAATACGTA | 625 | [[26](#ref26)] |
|  | *ah*-R | CGTGCTGGCAACAAAGGACAG |  |  |
| *aero* | *aero*-F | CACAGCCAATATGTCGGTGAAG | 326 | [[30](#ref30)] |
|  | *aero*-R | GTCACCTTCTCGCTCAGGC |  |  |
| *hly* | *hly*-F | CTATGAAAAAACTAAAAATAACTG | 1500 | [[29](#ref29)] |
|  | *hly*-R | CAGTATAAGTGGGGAAATGGAAAG |  |  |
| ***Salmonella* spp.** | |  |  |  |
| *invA* | *invA*-F | GTGAAATTATCGCCACGTTCGGGCAA | 284 | [[27](#ref27)] |
|  | *invA*-R | TCATCGCACCGTCAAAGGAACC |  |  |
| ***V. cholerae*** | |  |  |  |
| *ompW* | *ompW*-F | CACCAAGAAGGTGACTTTATTGTG | 588 | [[28](#ref28)] |
|  | *ompW*-R | GAACTTATAACCACCCGCG |  |  |
| *tcpA* | *tcpA*-F | CACGATAAGAAAACCGGTCAAGAG | 453 | [[31](#ref31)] |
|  | *tcpA*-R | CGAAAGCACCTTCTTTCACGTTG |  |  |
| *ctx* | *ctx*-F | CAGTCAGGTGGTCTTATGCCAAGAGG | 167 | [[32](#ref32)] |
|  | *ctx*-R | CCCACTAAGTGGGCACTTCTCAAACT |  |  |
| *hlyA* | *hlyA*-F | GGCAAACAGCGAAACAAATACC | 481 | [[33](#ref33)] |
|  | *hlyA*-R | CTCAGCGGGCTAATACGGTTTA |  |  |
