# Supplementary material for: Distribution of bacteria and antimicrobial resistance in retail Nile tilapia (Oreochromis spp.) as potential sources of foodborne illness
Source: PLoS One. 2024 Apr 2;19(4):e0299987. doi: 10.1371/journal.pone.0299987 (PMC10986973; doi:10.1371/journal.pone.0299987)
Supplement: S2 Table — (DOCX) [file pone.0299987.s002.docx]

**S2 Table. Primers used for detection of AMR genes of *A. hydrophila*, *Salmonella* spp., and *V. cholerae*.**

| **Gene** | **Primer** | **Oligonucleotide sequences** | **Product size (bp)** | **Reference** |
| --- | --- | --- | --- | --- |
| *bla*_TEM_ | *bla*_TEM_-F | GCGGAACCCCTATTT | 964 | [[61](#ref61)] |
|  | *bla*_TEM_-R | TCTAAAGTATATATGAGTAAACTTGGTCTGAC | |  |
| *bla*_SHV_ | *bla*_SHV_-F | TTCGCCTGTGTATTATCTCCCTG | 854 | [[62](#ref62)] |
|  | *bla*_SHV_-R | TTAGCGTTGCCAGTGYTG |  |  |
| *bla*_CTX-M_ | *bla*_CTX-M_-F | CGATGTGCAGTACCAGTAA | 585 | [[63](#ref63)] |
|  | *bla*_CTX-M_-R | AGTGACCAGAATCAGCGG |  |  |
| *bla*_NDM_ | *bla*_NDM_-F | GGTTTGGCGATCTGGTTTTC | 621 | [[64](#ref64)] |
|  | *bla*_NDM_-R | CGGAATGGCTCATCACGATC |  |  |
| *bla*_PSE_ | *bla*_PSE_-F | GCTCGTATAGGTGTTTCCGTTT | 575 | [[65](#ref65)] |
|  | *bla*_PSE_-R | CGATCCGCCGATGTTCCATCC |  |  |
| *bla*_OXA_ | *bla*_OXA_-F | ACACAATACATATCAACTTCGC | 813 | [[66](#ref66)] |
|  | *bla*_OXA_-R | AGTGTGTGTTTAGAATGGTGATC |  |  |
| *sul1* | *sul1*-F | CGGCGTGGGCTACCTGAACG | 433 | [[67](#ref67)] |
|  | *sul1*-R | GCCGATCGCGTGAAGTTCCG |  |  |
| *sul2* | *sul2*-F | CGGCATCGTCAACATAACCT | 721 | [[67](#ref67)] |
|  | *sul2*-R | TGTGCGGATGAAGTCAGCTC |  |  |
| *sul3* | *sul3*-F | TGTGCGGATGAAGTCAGCTC | 244 | [[67](#ref67)] |
|  | *sul3*-R | GCTGCACCAATTCGCTGAACG |  |  |
| *qnrA* | *qnrA*-F | AGAGGATTTCTCACGCCAGG | 580 | [[68](#ref68)] |
|  | *qnrA*-R | TGCCAGGCACAGATCTTGAC |  |  |
| *qnrB* | *qnrB*-F | GGMATHGAAATTCGCCACTG | 264 | [[68](#ref68)] |
|  | *qnrB*-R | TTTGCYGYYCGCCAGTCGAAC |  |  |
| *qnrS* | *qnrS*-F | GCAAGTTCATTGAACAGGGT | 428 | [[68](#ref68)] |
|  | *qnrS*-R | TCTAAACCGTCGAGTTCGGCG |  |  |
| *ermB* | *ermB*-F | AGACACCTCGTCTAACCTTCGCTC | 640 | [[69](#ref69)] |
|  | *ermB*-R | TCCATGTACTACCATGCCACAGG |  |  |
| *dfrA1* | *dfrA1*-F | GGAGTGCCAAAGGTGAACAGC | 367 | [[70](#ref70)] |
|  | *dfrA1*-R | GAGGCGAAGTCTTGGGTAAAAAC |  |  |
| *dfrA12* | *dfrA12*-F | TTCGCAGACTCACTGAGGG | 330 | [[71](#ref71)] |
|  | *dfrA12*-R | CGGTTGAGACAAGCTCGAAT |  |  |
| *catA* | *catA*-F | CCAGACCGTTCAGCTGGATA | 454 | [[71](#ref71)] |
|  | *catA*-R | CATCAGCACCTTGTCGCCT |  |  |
| *catB* | *catB*-F | CGGATTCAGCCTGACCACC | 461 | [[71](#ref71)] |
|  | *catB*-R | ATACGCGGTCACCTTCCTG |  |  |
| *cmlA* | *cmlA*-F | TGGACCGCTATCGGACCG | 641 | [[71](#ref71)] |
|  | *cmlA*-R | CGCAAGACACTTGGGCTGC |  |  |
| *strA* | *strA*-F | TGGCAGGAGGAACAGGAGG | 405 | [[71](#ref71)] |
|  | *strA*-R | AGGTCGATCAGACCCGTGC |  |  |
| *strB* | *strB*-F | GGCAGCATCAGCCTTATAATTT | 470 | [[72](#ref72)] |
|  | *strB*-R | GTGGATCCGTCATTCATTGTT |  |  |
| *tetA* | *tetA*-F | GGCGGTCTTCTTCATCATGC | 502 | [[67](#ref67)] |
|  | *tetA*-R | CGGCAGGCAGAGCAAGTAGA |  |  |
| *tetB* | *tetB*-F | CGCCCAGTGCTGTTGTTGTC | 615 | [[71](#ref71)] |
|  | *tetB*-R | CGCGTTGAGAAGCTGAGGTG |  |  |
| *tetD* | *tetD*-F | AAACCATTACGGCATTCTGC | 787 | [[73](#ref73)] |
|  | *tetD*-R | GACCGGATACACCATCCATC |  |  |
| *aadA1* | *aadA1*-F | CTCCGCAGTGGATGGCGG | 631 | [[71](#ref71)] |
|  | *aadA1*-R | GATCTGCGCGCGAGGCCA |  |  |
| *aadA2* | *aadA2*-F | CATTGAGCGCCATCTGGAAT | 500 | [[74](#ref74)] |
|  | *aadA2*-R | ACATTTCHCTCATCGCCGGC |  |  |
| *aac(3)IV* | *aac(3)IV*-F | GTGTGCTGCTGGTCCACAGC | 627 | [[75](#ref75)] |
|  | *aac(3)IV*-R | AGTTGACCCAGGGCTGTCGC |  |  |
| *aac(6’)-Ib-cr* | *aac(6’)-Ib-cr*-F | TTGCGATGCTCTATGAGTGGCTA | 482 | [[76](#ref76)] |
|  | *aac(6’)-lb-cr*-R | CTCGAATGCCTGGCGTGTTT |  |  |
| *qepA* | *qepA-*F | GCAGGTCCAGCAGCGGGTAG | 199 | [[77](#ref77)] |
|  | *qepA-*R | CTTCCTGCCCGAGTATCGTG |  |  |
| *floR* | *floR*-F | ATGGTGATGCTCGGCGTGGGCCA | 800 | [[78](#ref78)] |
|  | *floR*-R | GCGCCGTTGGCGGTAACAGACACCGTGA | |  |
| *mcr-1* | *mcr*-*1*-F | AGTCCGTTTGTTCTTGTGGC | 320 | [[79](#ref79)] |
|  | *mcr*-*1*-R | AGATCCTTGGTCTCGGCTTG |  |  |
| *mcr-2* | *mcr*-*2*-F | CAAGTGTGTTGGTCGCAGTT | 715 | [[79](#ref79)] |
|  | *mcr*-*2*-R | TCTAGCCCGACAAGCATACC |  |  |
| *mcr-3* | *mcr*-*3*-F | AAATAAAAATTGTTCCGCTTATG | 929 | [[79](#ref79)] |
|  | *mcr*-*3*-R | AATGGAGATCCCCGTTTTT |  |  |
| *mcr-4* | *mcr*-*4*-F | TCACTTTCATCACTGCGTTG | 1116 | [[79](#ref79)] |
|  | *mcr*-*4*-R | TTGGTCCATGACTACCAATG |  |  |
| *mcr-5* | *mcr*-*5*-F | ATGCGGTTGTCTGCATTTATC | 1644 | [[79](#ref79)] |
|  | *mcr*-*5*-R | TCATTGTGGTTGTCCTTTTCTG |  |  |
| **Integrons** |  |  |  |  |
| *int1* | *int1*-F | CCTGCACGGTTCGAATG | 497 | [[80](#ref80)] |
|  | *int1*-R | TCGTTTGTTCGCCCAGC |  |  |
| *int2* | *int2*-F | GGCAGACAGTTGCAAGACAA | 247 | [[80](#ref80)] |
|  | *int2*-R | AAGCGATTTTCTGCGTGTTT |  |  |
| *int3* | *int3*-F | CCGGTTCAGTCTTTCCTCAA | 155 | [[80](#ref80)] |
|  | *int3*-R | GAGGCGTGTACTTGCCTCAT |  |  |
| **Integrative Conjugative Elements (ICEs)** | | | | |
| *int*_SXT_ | *int*_SXT_-F | GCTGGATAGGTTAAGGGCGG | 592 | [[80](#ref80)] |
|  | *int*_SXT_-R | CTCTATGGGCACTGTCCACATTG |  |  |
| **QRDRs** | | | | |
| *gyr*A | *gyr*A-F | GCTGAAGAGCTCCTATCTGG | 436 | [[81](#ref81)] |
|  | *gyr*A-R | GGTCGGCATGACGTCCGG |  |  |
| *par*C | *par*C-F | GTACGTGATCATGGATCGTG | 390 | [[81](#ref81)] |
|  | *par*C-R | TTCCTGCATGGTGCCGTCG |  |  |
