# Supplementary material for: Distribution of bacteria and antimicrobial resistance in retail Nile tilapia (Oreochromis spp.) as potential sources of foodborne illness
Source: PLoS One. 2024 Apr 2;19(4):e0299987. doi: 10.1371/journal.pone.0299987 (PMC10986973; doi:10.1371/journal.pone.0299987)
Supplement: S3 Table — (DOCX) [file pone.0299987.s003.docx]

**S3 Table. *Salmonella* serovars isolated from Nile tilapia (*n* = 141)**

| **Serotype** | **No. of isolates (%)** | | | |
| --- | --- | --- | --- | --- |
|  | **Fish meat  (*n* = 74)** | **Liver and kidney  (*n* = 28)** | **Intestine  (*n* = 39)** | **Total  (*n* = 141)** |
| Aequatoria | 1 (1.4) | 0 (0) | 0 (0) | 1 (0.7) |
| Anatum | 1 (1.4) | 0 (0) | 0 (0) | 1 (0.7) |
| Anderlecht | 2 (2.7) | 0 (0) | 0 (0) | 2 (1.4) |
| Assinie | 0 (0) | 1 (3.6) | 0 (0) | 1 (0.7) |
| Athinai | 1 (1.4) | 0 (0) | 0 (0) | 1 (0.7) |
| Augustenborg | 1 (1.4) | 0 (0) | 0 (0) | 1 (0.7) |
| Braenderup | 2 (2.7) | 0 (0) | 3 (7.7) | 5 (3.5) |
| Bruebach | 1 (1.4) | 0 (0) | 0 (0) | 1 (0.7) |
| Bullbay | 0 (0) | 0 (0) | 1 (2.6) | 1 (0.7) |
| Catalunia | 2 (2.7) | 0 (0) | 0 (0) | 2 (1.4) |
| Derby | 0 (0) | 1 (3.6) | 0 (0) | 1 (0.7) |
| Enteritidis | 0 (0) | 0 (0) | 2 (5.1) | 2 (1.4) |
| Escanaba | 3 (4.1) | 2 (7.1) | 3 (7.7) | 8 (5.7) |
| Essen | 1 (1.4) | 0 (0) | 0 (0) | 1 (0.7) |
| Fallowfield | 1 (1.4) | 0 (0) | 2 (5.1) | 3 (2.1) |
| Fulda | 1 (1.4) | 0 (0) | 0 (0) | 1 (0.7) |
| Fyris | 1 (1.4) | 0 (0) | 0 (0) | 1 (0.7) |
| Garba | 1 (1.4) | 0 (0) | 0 (0) | 1 (0.7) |
| Gombe | 1 (1.4) | 0 (0) | 0 (0) | 1 (0.7) |
| Guerin | 0 (0) | 0 (0) | 1 (2.6) | 1 (0.7) |
| Haifa | 1 (1.4) | 0 (0) | 0 (0) | 1 (0.7) |
| Halle | 1 (1.4) | 0 (0) | 0 (0) | 1 (0.7) |
| Hato | 1 (1.4) | 0 (0) | 0 (0) | 1 (0.7) |
| Hoboken | 1 (1.4) | 0 (0) | 0 (0) | 1 (0.7) |
| Hohentwiel | 0 (0) | 0 (0) | 1 (2.6) | 1 (0.7) |
| II | 4 (5.4) | 1 (3.6) | 1 (2.6) | 6 (4.3) |
| IIIa | 1 (1.4) | 0 (0) | 0 (0) | 1 (0.7) |
| Infantis | 3 (4.1) | 3 (10.7) | 0 (0) | 6 (4.3) |
| Jukestown | 1 (1.4) | 0 (0) | 0 (0) | 1 (0.7) |
| Kentucky | 1 (1.4) | 1 (3.6) | 0 (0) | 2 (1.4) |
| Kibusi | 1 (1.4) | 0 (0) | 0 (0) | 1 (0.7) |
| Langeveld | 1 (1.4) | 0 (0) | 0 (0) | 1 (0.7) |
| Ligeo | 1 (1.4) | 0 (0) | 0 (0) | 1 (0.7) |
| Livingstone | 1 (1.4) | 0 (0) | 0 (0) | 1 (0.7) |
| Matopeni | 0 (0) | 0 (0) | 1 (2.6) | 1 (0.7) |
| Messina | 0 (0) | 0 (0) | 1 (2.6) | 1 (0.7) |
| Montevideo | 0 (0) | 2 (7.1) | 0 (0) | 2 (1.4) |
| Namibia | 1 (1.4) | 0 (0) | 0 (0) | 1 (0.7) |
| Neukoelln | 3 (4.1) | 2 (7.1) | 2 (5.1) | 7 (5.0) |
| Newlands | 1 (1.4) | 0 (0) | 0 (0) | 1 (0.7) |
| Nottingham | 0 (0) | 0 (0) | 1 (2.6) | 1 (0.7) |
| Ohlstedt | 1 (1.4) | 0 (0) | 0 (0) | 1 (0.7) |
| Orion | 0 (0) | 0 (0) | 1 (2.6) | 1 (0.7) |
| Oskarshamn | 2 (2.7) | 0 (0) | 0 (0) | 2 (1.4) |
| Othmarschen | 1 (1.4) | 0 (0) | 1 (2.6) | 2 (1.4) |
| Papuana | 0 (0) | 4 (14.3) | 3 (7.7) | 7 (5.0) |
| Paratyphi B | 1 (1.4) | 6 (21.4) | 2 (5.1) | 9 (6.4) |
| Plumaugat | 1 (1.4) | 1 (3.6) | 0 (0) | 2 (1.4) |
| Poona | 0 (0) | 0 (0) | 3 (7.7) | 3 (2.1) |
| Rissen | 1 (1.4) | 0 (0) | 0 (0) | 1 (0.7) |
| Saintpaul | 2 (2.7) | 4 (14.3) | 2 (5.1) | 8 (5.7) |
| Sandiego | 1 (1.4) | 0 (0) | 1 (2.6) | 2 (1.4) |
| Schwabach | 1 (1.4) | 0 (0) | 0 (0) | 1 (0.7) |
| Schwarzengrund | 1 (1.4) | 0 (0) | 0 (0) | 1 (0.7) |
| Shomolu | 1 (1.4) | 0 (0) | 0 (0) | 1 (0.7) |
| Singapore | 2 (2.7) | 0 (0) | 0 (0) | 2 (1.4) |
| Stanley | 2 (2.7) | 0 (0) | 2 (5.1) | 4 (2.8) |
| Sylvania | 1 (1.4) | 0 (0) | 0 (0) | 1 (0.7) |
| Tallahassee | 3 (4.1) | 0 (0) | 0 (0) | 3 (2.1) |
| Tomelilla | 1 (1.4) | 0 (0) | 0 (0) | 1 (0.7) |
| Trachau | 1 (1.4) | 0 (0) | 0 (0) | 1 (0.7) |
| Typhimurium | 1 (1.4) | 0 (0) | 0 (0) | 1 (0.7) |
| Uganda | 1 (1.4) | 0 (0) | 0 (0) | 1 (0.7) |
| Virchow | 6 (8.1) | 0 (0) | 1 (2.6) | 7 (5.0) |
| Vom | 0 (0) | 0 (0) | 1 (2.6) | 1 (0.7) |
| Warragul | 0 (0) | 0 (0) | 3 (7.7) | 3 (2.1) |
| **Total** | **74 (100.0)** | **28 (100.0)** | **39 (100.0)** | **141 (100.0)** |
