# Supplementary material for: Distribution of bacteria and antimicrobial resistance in retail Nile tilapia (Oreochromis spp.) as potential sources of foodborne illness
Source: PLoS One. 2024 Apr 2;19(4):e0299987. doi: 10.1371/journal.pone.0299987 (PMC10986973; doi:10.1371/journal.pone.0299987)
Supplement: S4 Table — (DOCX) [file pone.0299987.s004.docx]

**S4 Table**. **AMR patterns of *A. hydrophila* isolated from Nile tilapia (*n* = 26)**

| **Resistance pattern** | **No. of isolate (%)** | | | |
| --- | --- | --- | --- | --- |
|  | **Fish meat**  **(*n =* 7)** | **Liver and kidney  (*n* = 10)** | **Intestine**  **(*n* = 9)** | **Total**  **(*n =* 26)** |
| Susceptible | 0 (0) | 0 (0) | 0 (0) | 0 (0) |
| AMP-CHP-FFC-OTC-SMZ-STR-TET-TRI | 1 (14.3) | 1 (10.0) | 0 (0) | 2 (7.7) |
| AMP-FFC | 1 (14.3) | 2 (20.0) | 6 (66.7) | 9 (34.6) |
| AMP-FFC-OTC-SMZ-STR-TET-TRI | 0 (0) | 2 (20.0) | 0 (0) | 2 (7.7) |
| AMP-FFC-OTC-TET | 2 (28.6) | 3 (30.0) | 1 (11.1) | 6 (23.1) |
| AMP-FFC-OTC-TET-TRI | 2 (28.6) | 1 (10.0) | 0 (0) | 3 (11.5) |
| AMP-FFC-SMZ-TRI | 0 (0) | 1 (10.0) | 0 (0) | 1 (3.8) |
| AMP-FFC-STR | 0 (0) | 0 (0) | 1 (11.1) | 1 (3.8) |
| AMP-FFC-TRI | 1 (14.3) | 0 (0) | 1 (11.1) | 2 (7.7) |
| Total | 7 (100.0) | 10 (100.0) | 9 (100.0) | 26 (100.0) |

AMP, ampicillin; CHP, chloramphenicol; FFC, florfenicol; OTC, oxytetracycline; STR, streptomycin; SMZ, sulfamethoxazole; TET, tetracycline; TRI, trimethoprim
