# Supplementary material for: Distribution of bacteria and antimicrobial resistance in retail Nile tilapia (Oreochromis spp.) as potential sources of foodborne illness
Source: PLoS One. 2024 Apr 2;19(4):e0299987. doi: 10.1371/journal.pone.0299987 (PMC10986973; doi:10.1371/journal.pone.0299987)
Supplement: S5 Table — (DOCX) [file pone.0299987.s005.docx]

**S5 Table**. **AMR patterns of *Salmonella* isolated from Nile tilapia (*n* = 141)**

| **Resistance pattern** | **No. of isolates (%)** | | | |
| --- | --- | --- | --- | --- |
|  | **Fish meat  (*n* = 74)** | **Liver and kidney**  **(*n* = 28)** | **Intestine**  **(*n =* 39)** | **Total**  **(*n* = 141)** |
| Susceptible | 33 (44.6) | 7 (25.0) | 24 (61.5) | 64 (45.4) |
| AMP-CHP-CIP-ENR-FFC-OTC-OXO-TET | 2 (2.7) | 0 (0) | 0 (0) | 2 (1.4) |
| AMP-CHP-FFC | 0 (0) | 3 (10.7) | 1 (2.6) | 4 (2.8) |
| AMP-CHP-FFC-OTC-OXO-SMZ-STR-TET | 1 (1.4) | 0 (0) | 0 (0) | 1 (0.7) |
| AMP-CHP-FFC-OTC-OXO-TET | 2 (2.7) | 1 (3.6) | 7 (17.9) | 10 (7.1) |
| AMP-CHP-FFC-OTC-OXO-TET-TRI | 4 (5.4) | 1 (3.6) | 0 (0) | 5 (3.5) |
| AMP-CHP-OTC-OXO-TET-TRI | 1 (1.4) | 0 (0) | 0 (0) | 1 (0.7) |
| AMP-CHP-OTC-SMZ-TET | 1 (1.4) | 0 (0) | 0 (0) | 1 (0.7) |
| AMP-CHP-OTC-SMZ-TET-TRI | 0 (0) | 0 (0) | 1 (2.6) | 1 (0.7) |
| AMP-CHP-OXO | 3 (4.1) | 0 (0) | 0 (0) | 3 (2.1) |
| AMP-CHP-SMZ | 0 (0) | 1 (3.6) | 0 (0) | 1 (0.7) |
| AMP-CIP-ENR-OTC-OXO-TET | 1 (1.4) | 3 (10.7) | 2 (5.1) | 6 (4.3) |
| AMP-FFC | 0 (0) | 0 (0) | 1 (2.6) | 1 (0.7) |
| AMP-OTC-OXO-SMZ-STR-TET | 1 (1.4) | 0 (0) | 0 (0) | 1 (0.7) |
| AMP-OTC-SMZ-STR-TET | 3 (4.1) | 1 (3.6) | 0 (0) | 4 (2.8) |
| AMP-OTC-STR-TET | 0 (0) | 0 (0) | 1 (2.6) | 1 (0.7) |
| AMP-OXO-SMZ | 1 (1.4) | 0 (0) | 0 (0) | 1 (0.7) |
| AMP-SMZ | 0 (0) | 4 (14.3) | 1 (2.6) | 5 (3.5) |
| AMP-SMZ-STR-TET-TRI | 1 (1.4) | 0 (0) | 0 (0) | 1 (0.7) |
| AMP-SMZ-STR-TRI | 0 (0) | 1 (3.6) | 0 (0) | 1 (0.7) |
| AMP-SMZ-TET | 0 (0) | 1 (3.6) | 0 (0) | 1 (0.7) |
| AMP-TRI | 1 (1.4) | 0 (0) | 0 (0) | 1 (0.7) |
| CIP-ENR-OXO-SMZ-STR-TET | 0 (0) | 1 (3.6) | 0 (0) | 1 (0.7) |
| OTC-OXO-TET | 1 (1.4) | 0 (0) | 0 (0) | 1 (0.7) |
| OTC-SMZ-STR-TET | 1 (1.4) | 0 (0) | 0 (0) | 1 (0.7) |
| OTC-SMZ-TET | 0 (0) | 1 (3.6) | 0 (0) | 1 (0.7) |
| OTC-TET | 1 (1.4) | 0 (0) | 0 (0) | 1 (0.7) |
| OXO | 12 (16.2) | 1 (3.6) | 1 (2.6) | 14 (9.9) |
| OXO-SMZ | 1 (1.4) | 0 (0) | 0 (0) | 1 (0.7) |
| OXO-STR | 1 (1.4) | 0 (0) | 0 (0) | 1 (0.7) |
| SMZ | 1 (1.4) | 1 (3.6) | 0 (0) | 2 (1.4) |
| SMZ-STR-TET | 1 (1.4) | 0 (0) | 0 (0) | 1 (0.7) |
| SMZ-TRI | 0 (0) | 1 (3.6) | 0 (0) | 1 (0.7) |
| **Total** | **74 (52.5)** | **28 (19.9)** | **39 (27.7)** | **141 (100.0)** |

AMP, ampicillin; CHP, chloramphenicol; CIP, ciprofloxacin; ENR, enrofloxacin; FFC, florfenicol; OTC, oxytetracycline; OXO, oxolinic acid; STR, streptomycin; SMZ, sulfamethoxazole; TET, tetracycline; TRI, trimethoprim
