# Supplementary material for: Distribution of bacteria and antimicrobial resistance in retail Nile tilapia (Oreochromis spp.) as potential sources of foodborne illness
Source: PLoS One. 2024 Apr 2;19(4):e0299987. doi: 10.1371/journal.pone.0299987 (PMC10986973; doi:10.1371/journal.pone.0299987)
Supplement: S6 Table — (DOCX) [file pone.0299987.s006.docx]

**S6 Table**. **AMR patterns of *V. cholerae* isolated from Nile tilapia (*n* = 70)**

| **Resistance pattern** | **No. of isolates (%)** | | | |
| --- | --- | --- | --- | --- |
|  | **Fish meat  (*n* = 23)** | **Liver and kidney**  **(*n* =16)** | **Intestine**  **(*n* = 31)** | **Total**  **(*n* = 70)** |
| Susceptible | 7 (30.4) | 4 (25.0) | 12 (38.7) | 23 (32.9) |
| AMP | 8 (34.8) | 4 (25.0) | 6 (19.4) | 18 (25.7) |
| AMP-CHP | 0 (0) | 1 (6.3) | 0 (0) | 1 (1.4) |
| AMP-CHP-OTC-STR-TET | 0 (0) | 0 (0) | 1 (3.2) | 1 (1.4) |
| AMP-CHP-OTC-TET | 0 (0) | 0 (0) | 1 (3.2) | 1 (1.4) |
| AMP-OTC-OXO-STR-TET | 0 (0) | 0 (0) | 1 (3.2) | 1 (1.4) |
| AMP-OTC-TET | 3 (13.0) | 4 (25.0) | 3 (9.7) | 10 (14.3) |
| AMP-SMZ-TRI | 0 (0) | 1 (6.3) | 0 (0) | 1 (1.4) |
| AMP-STR | 0 (0) | 0 (0) | 1 (3.2) | 1 (1.4) |
| CHP-OXO-SMZ-TRI | 0 (0) | 1 (6.3) | 0 (0) | 1 (1.4) |
| CIP-ENR-OXO-SMZ-TRI | 0 (0) | 0 (0) | 1 (3.2) | 1 (1.4) |
| OTC-SMZ | 2 (8.7) | 0 (0) | 0 (0) | 2 (2.9) |
| OTC-SMZ-TET | 0 (0) | 0 (0) | 1 (3.2) | 1 (1.4) |
| SMZ | 3 (13.0) | 1 (6.3) | 4 (12.9) | 8 (11.4) |
| **Total** | **23 (32.9)** | **16 (22.9)** | **31 (44.3)** | **70 (100.0)** |

AMP, ampicillin; CHP, chloramphenicol; ENR, enrofloxacin; OTC, oxytetracycline; OXO, oxolinic acid; STR, streptomycin; SMZ, sulfamethoxazole; TET, tetracycline; TRI, trimethoprim
