# Supplementary material for: Distribution of bacteria and antimicrobial resistance in retail Nile tilapia (Oreochromis spp.) as potential sources of foodborne illness
Source: PLoS One. 2024 Apr 2;19(4):e0299987. doi: 10.1371/journal.pone.0299987 (PMC10986973; doi:10.1371/journal.pone.0299987)
Supplement: Attachment — Submitted filename: Comments.doc [file pone.0299987.s007.doc]

PONE-D-23-29694: Bacterial pathogens and antimicrobial resistance in retail Nile tilapia (Oreochromis spp.) as potential sources of foodborne illness

**General comments**

- Overall writing of the manuscript is good
- Several similar reports have been published in recent years, hence the work has limited novelty
- Focusing more on tilapia aquaculture without considering the farm source of the samples
- The overall prevalence of targeted bacterial species was low compared to previous reports. Authors could have easily highlighted the safety of the fish samples comparative to fish from other markets.
- The study observed no cholera causing vibrios among the isolates, which was not highlighted
- It is good that the study concluded suggesting better cooking methods to avoid possible food borne infection.
- It looks like the authors have ended the discussion abruptly.
- The conclusion part of the manuscript not corroborating with the results of the study
- As indicated by the authors the results of the study might be helpful to the Thailand’s National Action Plan of One Health Framework but dose-not advance the knowledge to the general reader.
- However, the study has required experimental design and the results have been analysed appropriately.
- Since the observed prevalence of the pathogenic bacteria are low in the study, change in the title may be considered.
- After carefully addressing the suggestions the manuscript may be considered for publication in the any of the regional journals

**Specific comments**

**Introduction**

Line no. 24-26: First two sentences are very generic in nature may be deleted from the abstract

Line no. 52-55: The bacteria isolated in the study and the identified AMR in the present study are not traced to farming system/production facilities. Hence, starting the manuscript with aquaculture intensification may not be appropriate. Instead highlights may be provided regarding the post harvest handling of fish and fishery products. Samples in the study are taken from markets and results suggest significant difference between the samples from fresh and supermarkets.

**Materials and methods**

Details of bacterial isolations are given in too details, unless any modifications in the standard protocols are made in the study, it is advisable to provide the references only.

**Results**

Line no. 285: fish were displayed without ice???

Table 1 & 2: Since the primers are are published earlier, mentioning the references may be sufficient

Table 4: This may be provided as supplementary information only, not in the main text

Fig 1 &2: Classifying the prevalence of AMR and the genes based on the tissue origin of the isolates may not be appropriate.

**Discussion**

Line No. 395-399: Since the farm source of the samples used in the study are not available, assuming that the AMR bacteria were due to unscientific use of antibiotics in the culture system may not be appropriate.

Line No. 403-405: Correlating the presence of resistance gene against particular antibiotic in an isolate to its use in the farming system without actual information on the farm usage data may not be appropriate.

Line No. 459-461: Since not data related to farming activity was collected in the study, it may not be appropriate to suggest the observations are due to usage of antibiotics in culture operations.

Line No. 459-464: first sentence indicate frequent use of antibiotics in farms while the next sentence suggest need for better handling practices to reduce the cross contamination. These two sentences are unrelated.

**References:** Total number of references cited by be reduced substantially
