# Supplementary material for: Distribution of bacteria and antimicrobial resistance in retail Nile tilapia (Oreochromis spp.) as potential sources of foodborne illness
Source: PLoS One. 2024 Apr 2;19(4):e0299987. doi: 10.1371/journal.pone.0299987 (PMC10986973; doi:10.1371/journal.pone.0299987)
Supplement: Attachment — Submitted filename: Response to Reviewers letter 260124.docx [file pone.0299987.s009.docx]

**Manuscript ID:** PONE-D-23-29694R1: Distribution of bacteria and antimicrobial resistance in retail Nile tilapia (Oreochromis spp.) as potential sources of foodborne illness

**Editor’s comments**

| **Comment** | **Response to reviewer** |
| --- | --- |
| 1. Please highlight the novelty of this study vs other published studies in the area.  Please also address other comments of the reviewer. | 1. The sentence relating to the novelty of this study is modified in Line 130-135.  “Few studies have conducted investigations into AMR pathogens among retail tilapia intended for human consumption, and the risk of AMR transmission from tilapia consumption has been insufficiently documented in Thailand.” |
| 2. If applicable, we recommend that you deposit your laboratory protocols in [protocols.io](http://protocols.io/) to enhance the reproducibility of your results. | All bacterial determination and confirmation, antimicrobial susceptibility testing, and detection of AMR and virulence genes in this study followed standard methods, which are available online. However, specific laboratory protocols are available upon request. |

**Reviewer’s comments**

| **Comment** | **Response to reviewer** |
| --- | --- |
| 1. Did the standard cultures of *A. hydrophila*, *V. cholerae* and *Salmonella* used in the study? if so please specify. | 1. The standard cultures of *A. hydrophila*, *V. cholerae*, *and Salmonella* were indicated in materials and methods section (Line 212-213).  “The reference stains used in this study were *A. hydrophila* DMST 2798, *Salmonella* Enteritidis DMST 15676, and *V. cholerae* DMST 2873.” |
| 2. While writing the name of the genes, do not italicize the subunit only the name of the gene should be italic for ex. gyr A where gyr need to be italic not A | 2. The errors have been corrected throughout manuscript included Table2. |
| 3. Highlight the novelity in the introduction section because we can find similar kind of work in the database <https://doi.org/10.3389/frabi.2023.1156258> 10.7717/peerj.14896 | 3. The novelty of this study is indicated in Line 130-135.  “Few studies have conducted investigations into AMR pathogens among retail tilapia intended for human consumption, and the risk of AMR transmission from tilapia consumption has been insufficiently documented in Thailand.” |
