# Supplementary material for: Distribution of bacteria and antimicrobial resistance in retail Nile tilapia (Oreochromis spp.) as potential sources of foodborne illness
Source: PLoS One. 2024 Apr 2;19(4):e0299987. doi: 10.1371/journal.pone.0299987 (PMC10986973; doi:10.1371/journal.pone.0299987)
Supplement: Attachment — Submitted filename: Reviewer comments.docx [file pone.0299987.s008.docx]

1. The authors would have mentioned the details of positive controls used for the study.
2. What is the rationale behind the selection of three pathogens? The authors should emphasize this in the manuscript.
3. Why have authors restricted to only a few virulence genes in 3 pathogens? Especially in *Salmonella* and *Aeromonas hydrophilla*Line 198 rnase should be written as RNAase
4. Few typographical errors in the manuscript need to be taken care
5. It is better if the Authors use h or hr or hours in the manuscript
